# Electrical impedance tomography monitoring in adult ICU patients: state-of-the-art, recommendations for standardized acquisition, processing, and clinical use, and future directions

**DOI:** 10.1186/s13054-024-05173-x

**Published:** 2024-11-19

**Authors:** Gaetano Scaramuzzo, Bertrand Pavlovsky, Andy Adler, Walter Baccinelli, Dani L. Bodor, L. Felipe Damiani, Guillaume Franchineau, Juliette Francovich, Inéz Frerichs, Juan A. Sánchez Giralt, Bartłomiej Grychtol, Huaiwu He, Bhushan H. Katira, Alette A. Koopman, Steffen Leonhardt, Luca S. Menga, Amne Mousa, Mariangela Pellegrini, Thomas Piraino, Paolo Priani, Peter Somhorst, Elena Spinelli, Claas Händel, Fernando Suárez-Sipmann, Jantine J. Wisse, Tobias Becher, Annemijn H. Jonkman

**Affiliations:** 1 Department of Translational Medicine, University of Ferrara, Ferrara, Italy; 2 Medical Intensive Care Unit, Vent’Lab, Angers University Hospital, University of Angers, 4 Rue Larrey, 49933 Angers Cedex 9, France; 3 Systems and Computer Engineering, Carleton University, Ottawa, Canada; 4 Netherlands eScience Center, Amsterdam, The Netherlands; 5 Facultad de Medicina, Escuela de Ciencias de La Salud, Pontificia Universidad Catolica de Chile, Santiago, Chile; 6 Service de Medecine Intensive Reanimation, Centre Hospitalier Intercommunal de Poissy-Saint-Germain-en-Laye, Poissy, France; 7 Department of Intensive Care Medicine, Erasmus Medical Center, Rotterdam, The Netherlands; 8 Department of Anesthesiology and Intensive Care Medicine, University Medical Center Schleswig-Holstein, Campus Kiel, Kiel, Germany; 9 Intensive Care Unit, Hospital Universitario La Princesa, Madrid, Spain; 10 State Key Laboratory of Complex Severe and Rare Diseases, Department of Critical Care Medicine, Peking Union Medical College Hospital, Peking Union Medical College, Chinese Academy of Medical Sciences, Beijing, China; 11 Department of Pediatrics, Washington University, St. Louis, MO USA; 12 Division of Paediatric Critical Care Medicine, Department of Paediatrics, Beatrix Children’s Hospital, University Medical Center Groningen, Groningen, The Netherlands; 13 Chair for Medical Information Technology, RWTH Aachen University, Aachen, Germany; 14 Interdepartmental Division of Critical Care Medicine, Keenan Research Centre, Li Ka Shing Knowledge Institute, St. Michael’s Hospital, University of Toronto, Toronto, Canada; 15 Department of Intensive Care Medicine, Amsterdam UMC, Amsterdam, The Netherlands; 16 Hedenstierna Laboratory, Department of Surgical Sciences, Uppsala University, Uppsala, Sweden; 17 Department of Anesthesia, Division of Critical Care, McMaster University, Hamilton, ON Canada; 18 Department of Anesthesia, Critical Care and Emergency, Fondazione IRCCS Ca’ Granda Ospedale Maggiore Policlinico, 20122 Milan, Italy; 19 CIBER de Enfermedades Respiratorias, Instituto de Salud Carlos III, Madrid, Spain; 20 Dept. Intensive Care Volwassenen, Erasmus Medical Center, Room Ne-403, Dr. Molewaterplein 40, 3015 GD Rotterdam, The Netherlands

**Keywords:** Electrical impedance tomography, Mechanical ventilation, Respiratory monitoring, Signal processing, Ventilation distribution, Lung perfusion

## Abstract

Electrical impedance tomography (EIT) is an emerging technology for the non-invasive monitoring of regional distribution of ventilation and perfusion, offering real-time and continuous data that can greatly enhance our understanding and management of various respiratory conditions and lung perfusion. Its application may be especially beneficial for critically ill mechanically ventilated patients. Despite its potential, clear evidence of clinical benefits is still lacking, in part due to a lack of standardization and transparent reporting, which is essential for ensuring reproducible research and enhancing the use of EIT for personalized mechanical ventilation. This report is the result of a four-day expert meeting where we aimed to promote the consistent and reliable use of EIT, facilitating its integration into both clinical practice and research, focusing on the adult intensive care patient. We discuss the state-of-the-art regarding EIT acquisition and processing, applications during controlled ventilation and spontaneous breathing, ventilation-perfusion assessment, and novel future directions.

**Supplementary Information:**

The online version contains supplementary material available at 10.1186/s13054-024-05173-x.

## Introduction

Imaging plays a crucial role in the diagnosis and monitoring of critically ill patients. Among others, electrical impedance tomography (EIT) has gained popularity as a safe, non-invasive and validated bedside technique for real-time continuous evaluation of the ventilation and perfusion distribution [1, 2]. Given its high temporal resolution, ability to show regional ventilation/perfusion and track changes over time, EIT may be particularly valuable in mechanically ventilated patients [1–5]. EIT improves our physiological understanding of respiratory failure at bedside (e.g., with hypoxemia, pulmonary derecruitment, or potentially patient self-inflicted lung injury—P-SILI). It can also monitor and assess the dynamic response to maneuvers (e.g. titration of positive end-expiratory pressure (PEEP) [3], prone positioning [4]) and enables investigating specific pulmonary conditions over time.

Hence, EIT application may be crucial in point-of-care evaluation of lung physiology and delivering of individualized mechanical ventilation. Despite its potential, clear evidence of clinical benefits is still lacking [1, 5]. This may be related to technical barriers and a lack of standardization in data processing and in the interpretation of analyses, which is key to allow successful clinical integration [6].

To evaluate the state-of-the-art of EIT monitoring and to stimulate standardized experimental use and thereby to promote a successful implementation of EIT in clinical practice, a four-day expert meeting was held in the spring of 2024 in Leiden (The Netherlands) to discuss how to best acquire, process, analyze and interpret EIT data, both in a clinical and scientific context. The expert group comprised professionals with different clinical and/or scientific expertise in EIT, including medical doctors, technical physicians, respiratory therapists and biomedical engineers. This paper summarizes the insights gained from this meeting and provides recommendations on five areas of interest: (1) EIT acquisition, (2) EIT signal and image processing, (3) applications during controlled ventilation, (4) applications during spontaneous breathing, and (5) ventilation-perfusion assessment, focusing on adult patients in the intensive care unit (ICU). Novel future directions of EIT are also discussed.

## EIT acquisition

**Fig. 1:**
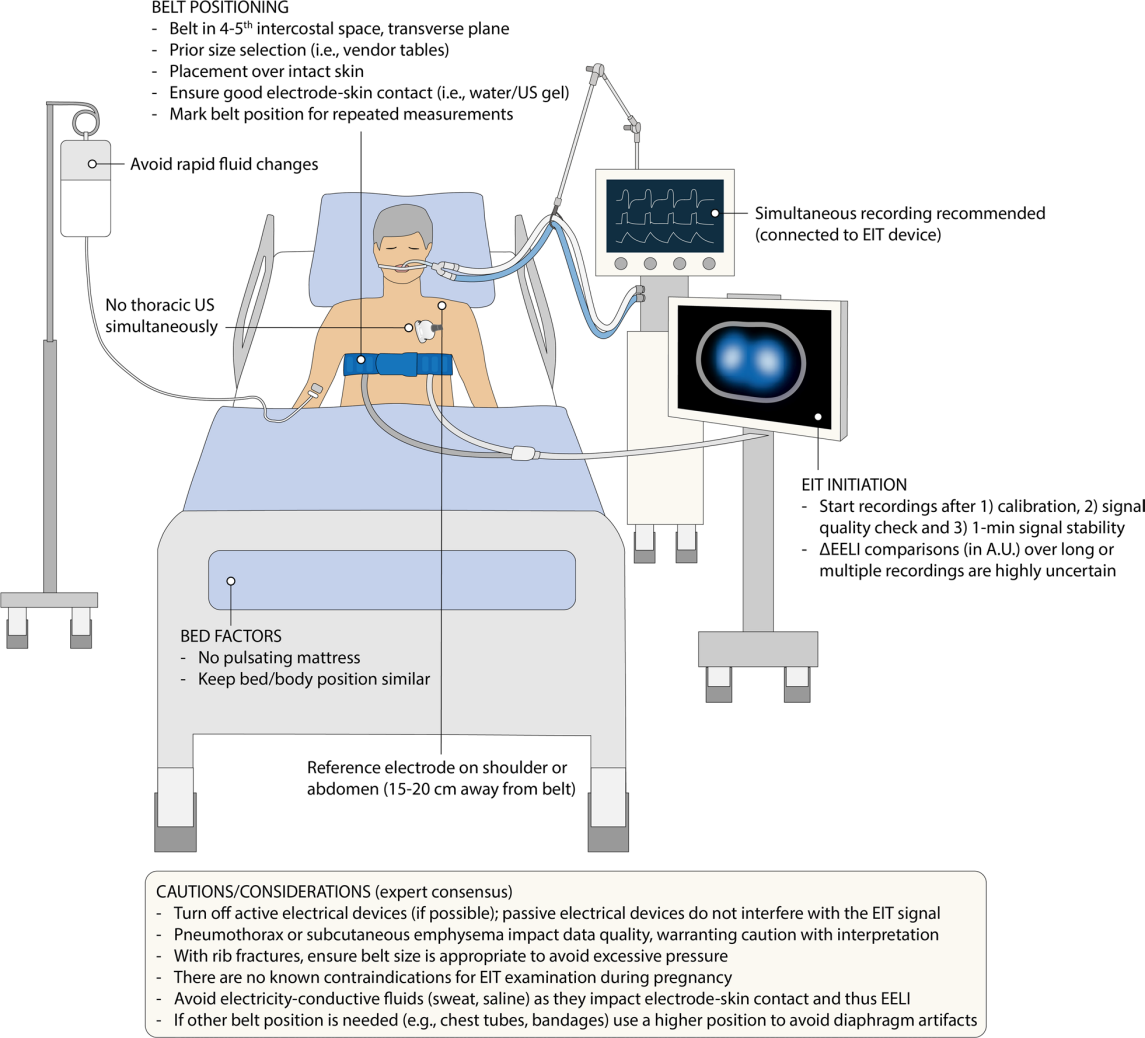
EIT acquisition and best practices. Abbreviations: A.U., arbitrary units; EELI, end-expiratory lung impedance; EIT, electrical impedance tomography; US, ultrasound

Best practices are summarized in Fig. 1.

### Electrode belt placement and EIT initiation

For in-depth technical principles of EIT, we refer to Frerichs et al. [2]. To date, indications and contraindications for EIT use are not necessarily evidence-based, but rather resulting from expert consensus. The first step to obtain a reliable measurement is the correct positioning of the electrode belt. The belt is typically positioned transversely between the 4th and 5th intercostal space [2], measured in the parasternal line. Positioning of the belt too low could result in artifacts from diaphragm movement and inaccurate values of tidal impedance variation (TIV) [7]. Conversely, placement of the belt too high may induce an erroneous estimation of ventilated areas, especially regarding dorsal regions [8–10]. Belt rotation should also be avoided, as it will affect the reconstructed image [11]. Belt plane orientation is also important: an obliquely placed belt (i.e., dorsal part of the belt placed more cranially than its ventral part), will result in an underrepresentation of the dorsal lung and, hence, erroneous interpretation of dorsal hypoventilation/collapse. A transverse plane is suggested for standard EIT monitoring.

The presence of chest tubes, bandages, wounds or skin burns may hinder correct belt placement. EIT devices generally work properly in the absence of 1 or 2 electrode pairs (for 16 and 32 electrode belts, respectively). If the conventional belt position is not possible, a higher belt placement is recommended. In the presence of rib fractures, careful selection of belt size and position is important to avoid any excessive pressure on the chest.

Belt size can be selected according to predefined tables based on the half-chest perimeter (measured from sternum to spine) [2]. Appropriate size selection prior to the measurement allows optimal inter-electrode spacing (overlapping electrodes should be avoided) and skin contact, minimizes the belt’s potential impact on chest wall compliance, and reduces waste with disposables. Electrode-to-skin contact can be improved by applying water, crystalloid fluid, ultrasound gel, or device-specific contact agents, according to the manufacturer. If required for the specific device, the reference electrode should be placed 15–20 cm from the belt’s plane, ideally on the abdomen or shoulder.

After belt positioning, EIT recordings should be started after device calibration (when possible), signal quality check, and a period of signal stability (at least 1 min recommended). Checking for stability after recordings also helps to ensure reliability of EIT measurements.

### Synchronized recordings

Simultaneous measurement of EIT with other physiological signals (i.e., respiratory waveforms, esophageal pressure, ECG) facilitates analyses and interpretation. Recording data on a single device is recommended, e.g., using a device-specific connection or flow/pressure sensor attached to the EIT machine. Absolute synchrony between sources can never be guaranteed due to acquisition and processing delays. However, if corresponding breaths are identified in different signals/sources, breath-by-breath comparison is possible. In this case, performing breath-hold maneuvers with different durations during the acquisition serves as a useful reference for (offline) synchronization.

### Acquisition challenges and considerations

Several factors need careful attention for reliable EIT acquisition:

- Negative inspiratory impedance changes: Diaphragm movement, pleural effusion, pneumothorax, external chest compressions or coughing may induce negative TIV [8]. While some of them are generally considered artifacts (e.g., diaphragmatic movement), others (e.g., pleural effusion, pneumothorax) may have clinical value [12, 13]. Of note, the presence of pneumothorax, pleural effusion and subcutaneous emphysema may impact TIV signal quality, making meaningful interpretations difficult.
- End-expiratory lung impedance (EELI) stability: Changes in end-expiratory lung impedance (∆EELI), i.e., due to PEEP adjustments, are frequently used to track changes in end-expiratory lung volume (∆EELV) as both closely correlate [14–17]. However, EELI may also change because of artifacts, such as pulsation of inflatable mattresses, degradation of contact agent, patient movements (active or passive, including bed angle), rapid changes in fluid balance, and belt repositioning [18–23]. ∆EELI calculations over long or multiple recordings should therefore be avoided. Furthermore, since device-specific calibrations and measurements are in arbitrary units (A.U.), absolute EELI and ∆EELI values cannot be compared among different patients. Short-term within-patient EELI changes can be compared, when mitigating artifacts. For between-patient comparisons, absolute EELI cannot be used, even after calibration. As for between-patient comparisons in ∆EELI, a patient-specific calibration (i.e., conversion from A.U. to mL via e.g., spirometry [24] or by recording tidal volumes on the ventilator [25]) has been used, but point-calibrations depend on the lung condition in that setting and therefore should be repeated if lung tissue properties change. Alternatively, the relative percentage change in EELI as compared to TIV can be used.
- Electrical-based interference: Active pacemakers/ICD are generally considered a safety contraindication for EIT use, but there is no evidence that modern EIT devices could negatively influence their functioning. However, vice-versa, active pacemakers/ICD can interfere with the EIT recording (creating artifacts in the EIT signal) unless a special artifact filter is enabled on the EIT device. Respiratory electromyography (e.g., for neurally-adjusted ventilator assist (NAVA)) is a passive measurement and therefore does not interfere with the EIT signal if both are properly positioned. In contrast, the EIT signal could potentially influence electromyography recordings. There is no data regarding interactions between EIT signals and respiratory muscle stimulation (e.g., phrenic nerve stimulation).
- Repeated measures over long time periods: Longitudinal acquisition is exposed to potential errors, since both belt position and patient factors may differ between acquisition time-points. Marking the belt location with a skin marker can be a reasonable option when repeated measures are planned, to ensure comparability among recordings. While EELI comparisons over multiple recordings should be avoided (see above), the evaluation of tidal ventilation distribution (e.g., % of TV in ROI4) is less affected by longitudinal acquisition artifacts if belt position is preserved.

## EIT signal processing

EIT signal processing generally involves: (1) filtering to extract a clean respiratory and/or cardiovascular signal, (2) selection of functional lung regions (i.e., lung segmentation), and/or (3) selection of regions of interest (ROI), if used. Each step alters the EIT signal differently and thus affects the computation and interpretation of parameters, warranting clear reporting.

### Signal filtering

EIT acquires voltage data subsequently reconstructed to impedance data and visualized as two-dimensional images (generally 32 × 32 pixels) containing information about lung ventilation and perfusion, heart action, but also disturbances/noise from (un)known sources. Most EIT applications require signal filtering prior to analysis, which can be performed at different stages: pre-reconstruction (filtering voltage data), directly post-reconstruction (filtering pixel impedance data), or after summing pixel impedances to a global or regional tidal impedance signal. Filtering voltage data requires intimate knowledge of the EIT hardware and raw data access, making this option frequently impractical.

Current devices mostly use frequency filters for processing and visualizing data on-device. The cardiovascular signal often is removed with low-pass filters, with a cutoff frequency between the respiratory rate and heart rate. However, this also removes harmonics (i.e., higher frequency details) of the respiratory signal of interest, thus introducing alterations in amplitude, EELI and timing [26].

More sophisticated filters can be considered during offline processing (depending on the use-case), requiring export of unfiltered pixel impedance data and/or disabling filters on-device (Table 1). As phase differences between pixel-level signals might influence the resulting summed impedance signal, we recommend filtering pixel impedance data before summation.

**Table 1:** Methods for processing EIT signals

Method	Specifics	Common pitfalls	Best practices/comments
Raw signal (unfiltered)	No filter is applied. Data is as obtained from the device	Manufacturers (can) apply filters during acquisition, reconstruction and before exporting data	Ensure raw data is actually unfiltered before stating so
Ensemble averaging	All features (e.g., breaths or cardiovascular wave) in (part of) a signal are detected and then averaged at a synchronized time. It reduces out-of-sync signals by averaging. The resulting feature can be analysed or used as a template for further detection of features [125] Note that ensemble averaging is not a filtering method per se but should result in a reconstructed signal free of disturbances	Ensemble averaging requires enough detected instances of a feature to obtain a reliable average. It improves the signal-to-noise ratio of the signal by the square root of the number of repetitions. This technique is therefore not applicable for short sections of a signal When the cardiovascular and respiratory signals share a harmonic, i.e., the heart rate is a multiple of the respiratory rate or vice versa), ensemble averaging might not remove unwanted features Since e.g., cardiovascular noise can shift the position of the minimum and maximum values of breaths, these should not be used to detect the temporal position of breaths	Preferable method when highly detailed information, e.g., the shape of the expiratory phase of a breath, is required Detection of the temporal position of breaths in EIT data should involve the entire breath shape, not just the start, end or peak of the breath. Alternatively, external signals, like simultaneously recorded and synchronised flow data can be used to identify the temporal position of breaths [126]
Frequency filters	Various frequency filters exist: low-pass filter, high-pass filter, band-pass filter, or band-stop filter. Frequency filters are mostly designed as Butterworth or Chebyshev type-II filters, which are infinite impulse response (IIR) filters. In general, finite impulse response (FIR) filters are preferred for better stability and phase response [127]	Most signals, like the respiratory signal, have a base frequency (band) and harmonics. Removal of the base frequency band does not remove the harmonics. Removal of the harmonics changes the shape of the signal, including the exact amplitude (relevant for TIV, small changes in EELI, etc.) and timing (relevant for I:E-ratio, asynchrony, etc.)	As these filters introduce a phase delay (FIR often delays frequency components by the same amount; the delay introduced by IIR is often variable among frequencies), they should be applied twice (forward + backward) to correct for distortions [128]
*Low-pass filter*	To remove high-frequency information like noise, cardiovascular signal, and some movement artifacts	If the cut-off frequency is too low, values might be altered significantly, affecting the results of the analyses	Use a low-pass filter to remove any very high frequency noise (above several multiples of the respiratory rate) Low-pass filters can be used to remove the cardiac signal [129] if one accepts losing high frequency components of the respiratory signal
*High-pass filter*	To remove low-frequency information like drift (e.g., due to changes in electrode conductivity), changes in whole-subject conductivity (e.g., due to changes in fluid status) and slow changes in EELI	High-pass filters remove any signal due to changes in EELI, and therefore is not recommended for use when studying changes in EELI	High-pass filters can be applied when the signal average is assumed constant over time, e.g., for calculation of tidal variations or breath averages
*Band-pass filter*	A band pass filter is a combination of a low-pass and high-pass filter, leaving only the signal with the selected frequency band [130–133]	See general comments for frequency filters	Band-pass filters can be used to extract features with a known small frequency band, like the effects of high frequency oscillatory ventilation [134]
*Band-stop filter*	Band-stop (or notch) filters can remove a specific band of frequencies, like the respiratory signal (for analysis of the cardiovascular signal)	See general comments for frequency filters	Band-stop filters should be used to filter out disturbances with a known frequency. Consider removing the harmonics as well Can be a very effective filter to remove cardiovascular and other high frequency noise from a stable signal when applied to the cardiovascular frequency and its harmonics in combination with a low-pass and high-pass filter [26]
Principal component analysis (PCA)	PCA is aimed at extracting components, a combination of different pixels, that contain the most variance. They have mainly been used to separate the respiratory and cardiovascular signal [127, 135]		
Empirical Mode Decomposition	EMD extracts distinct modes from a signal. Each mode represents a part of the signal within a specific frequency band. A signal, e.g., respiratory or cardiovascular, can be reconstructed from a subset of the modes [26, 135, 136]	The number of modes resulting from EMD is unknown before and depends on the complexity of the signal. Signals from different sources or at different times might require a different mode subset for reconstruction of a signal	The performance of EMD improves with masking, a technique that improves extraction of desirable modes by overlaying a known signal with a similar frequency. The performance can be significantly improved by using complete ensemble empirical mode decomposition with adaptive noise [136]
Discrete Wavelet Transform (DWT)	DWT deconstructs a signal into several bands with increasing frequency content based on wavelets. Signals can be reconstructed by selecting one or multiple frequency bands [26, 137]	DWT can remove drift, step-like and spike-like disturbances [137]. Although these methods seem to work in simulated data, it is unknown whether the offset in the resulting signal can be used for EELI-based analyses	The performance of DWT depends on wavelet selection and decomposition levels. Daubechies and Symlet type wavelets with high order perform well in maximal overlap discrete wavelet transform (MODWT), a modified version of DWT [26]

### Lung segmentation and region of interest (ROI) selection

*Lung segmentation* Since the EIT image comprises the chest portion within the belt plane (e.g., lung, chest wall, air outside the lungs, pleural effusion), lung segmentation aims to identify only lung tissue, thereby excluding non-parenchymal tissue (Additional file 1) [27]. To date, except from reconstruction of lung contouring based on patient’s anthropometric characteristics (i.e., as in the LuMon™ system, Sentec), extracting the total lung contour (including ventilated and non-ventilated lung tissue) directly from the EIT signal is impossible. Functional lung contouring is usually based on a TIV cut-off (typically a percentage of the maximum pixel’s TIV within the analyzed tidal image, e.g., 10–15%) [27, 28] which represents a reasonable choice but excludes non-functional lung regions (e.g., atelectasis). Elimination of the heart region when defining the lung contour should be carefully considered [29].

*ROIs* A region of interest (ROI) represents a subset of pixels. ROI definition is fundamental to exploit regional analysis of lung behavior as it allows inter-regional comparison within the lung area (e.g., ventral vs. dorsal), for instance to assess the effects of PEEP or positional strategies in term of ventilation re-distribution [30, 31]. ROIs can characterize the spatial ventilation heterogeneity using horizontal layers, vertical layers or quadrants. ROI analysis can be implemented to different images, therefore leading to different results:

- Whole EIT image ROIs: The whole EIT image is considered (ventilated + non-ventilated area); this allows the inclusion of hypo-ventilated areas, increasing the discriminatory power of ventilation inhomogeneity [27]. ROIs are defined based on the division of the pixel matrix, often in equidimensional regions (e.g., quadrants, or 4 regions of each 8 pixel rows). However, it is not uncommon for the most dependent ROI to exhibit little or no TIV, since the exact position of the lungs is affected by the patient’s anatomy, device-specific image reconstruction models and belt positioning. This could make the interpretation of the dependent regions difficult.
- Functional EIT image ROIs: Only the segmented lung area is considered (functional lung contour), which can ease the comparison between dependent and non-dependent lung regions. Geometrical ROIs (layers/quadrants) can be applied to this functional lung region. However, the definition is purely functional, and the so-defined dorsal region may be different from the dorsal region defined with e.g., CT scan. Recently, a new method for ROI selection was described, where on average, each ROI represents an equal contribution to the TIV of the full EIT recording of interest [19, 32]. This physiological approach is consistent with using the center of ventilation to separate the ventral and dorsal lung but includes multiple layers [32].

## Clinical applications: controlled ventilation

Several indices were developed to describe the global, spatial and temporal ventilation distribution, based on the pixel and/or regional variations of impedance (Table 2). When used in conjunction with specific ventilator procedures, EIT provides regional information that may be missed by global respiratory mechanics monitoring alone.

**Table 2:** EIT parameters and their applications in different ventilation conditions, for clinical and/or scientific purposes (as per current evidence) as well as bedside availability has been noted

	Ventilatory mode			Purpose		Availability		Comments
	Controlled	Assisted	Non-intubated	Clinical	Research	Bedside	Offline	
VT distribution	+	+	+	+	+	+	+	
EELI distribution	+	+	+	+ / −	+	+	+	EELI dependent
Dorsal fraction of ventilation	+	+	+	+	+	+	+	
CoV	+	+	+	+	+	+	+	
Global Inhomogeneity	+ / −	+ / −	+ / −	+ / −	+ / −	−	+	
OD-CL	+	+ / −	+ / −	+	+	+	+	Needs standardization
Regional PV-curve	+	−	−	+ / −	+	+ / −	+	Low-flow insufflation
RVD, RVDi	+	−	−	+ / −	+	+	+	Low-flow insufflation
Silent spaces	+	+	+ / −	+	+	+	+	
Time constant	+	+ / −	+ / −	−	+	−	+	
EIT-based R/I ratio	+	+ / −	+ / −	+ / −	+	+	+	EELI dependent
$\dot{\text{V}}/{\text{Q}}$ matching	+	+ / −	+ / −	+ / −	+	+ / −	+	Needs saline bolus
Volume estimation in SB	NA	NA	+	−	+	+ / −	+	
Pendelluft detection	+	+	+	+ / −	+	+ / −	+	

+ , the expert panel recommend the use of this parameter in this condition; + / − , the expert panel cannot state any recommendation in this condition; − , the expert panel does not recommend the use of this parameter in this condition

*Abbreviations:* NA, not applicable; CoV, center of ventilation; EELI, end-expiratory lung impedance; CL, lung collapse; OD, overdistension; PV-curve, pressure–volume curve; R/I ratio, recruitment-to-inflation ratio; RVD(i), regional ventilation delay (index); SB, spontaneous breathing; VT, tidal volume; $\dot{\text{V}}/{\text{Q}}$, ventilation-perfusion

### EIT to titrate positive end-expiratory pressure (PEEP)

The overdistension and lung collapse (OD-CL) method [3] has been most used in clinical studies [33–39] to assess the impact of PEEP on regional ventilation distribution and titrate PEEP. It involves assessing regional compliance changes, commonly during a decremental PEEP trial: compliance loss towards higher PEEP is interpreted as overdistension (OD), whereas compliance loss towards lower PEEP represents collapsed lung (CL). The optimal PEEP is then usually considered the one found at the intersection of the collapse and overdistension curves [3]: the PEEP that jointly minimizes both phenomena. This also assumes that collapse and overdistension contribute equally to ventilator-induced lung injury (VILI), but in some conditions this may not be true [40]. Nevertheless, a recent meta-analysis demonstrated that this strategy for individualizing PEEP improves respiratory mechanics and potentially outcomes in ARDS [36].

The OD-CL method requires driving pressure measurement to estimate regional compliance and should therefore be performed in volume-controlled ventilation (with inspiratory pause > 0.5 s and no intrinsic PEEP), when driving pressure can be automatically collected by the EIT machine or is added by the operator in post-processing. Otherwise, pressure-controlled mode, with constant support level and with sufficient time for equilibration between alveolar and airway pressures at end-inspiration and end-expiration (no-flow state), should be used.

**Fig. 2:**
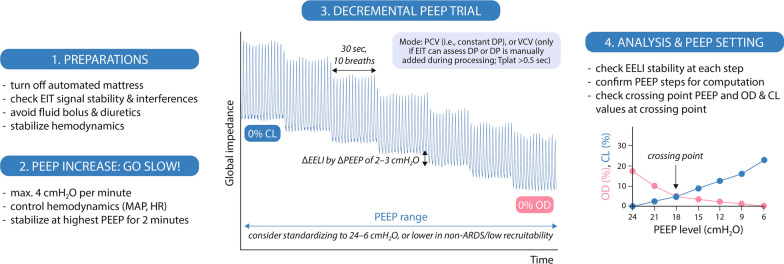
EIT-based PEEP titration: step-by-step recommendations. The PEEP selection is generally made at the crossing point of the OD and CL curves; if the crossing point is between two PEEP levels, values are usually rounded up to the nearest integer. Abbreviations: ARDS, acute respiratory distress syndrome; DP, driving pressure; EELI, end-expiratory lung impedance; EIT, electrical impedance tomography; HR, heart rate; CL, lung collapse; MAP, mean arterial pressure; OD, overdistension, PEEP, positive end-expiratory pressure; PCV, pressure-controlled ventilation; Tplat, time (duration) of plateau pressure; VCV, volume-controlled ventilation

Importantly, since *relative* regional compliance changes are computed and thus *relative* collapse and overdistension (i.e., highest PEEP = 0% collapse, lowest PEEP = 0% overdistension), the chosen PEEP range affects results [41]. Any remaining collapse at the highest PEEP level (as per CT scan) is not visible on EIT. Hence, the optimal PEEP reflects the PEEP able to jointly minimize relative collapse and overdistension only *in the explored PEEP range*. To improve reliability and inter-patient comparisons, a standardized PEEP window (e.g., from 24 to 6 cm H_2_O, as previously done [35]) is preferable, especially for clinical trials. If a narrower range is applied, especially in non-ARDS or poorly recruitable lungs, a crossing point close to the boundaries (e.g., < 3 cm H_2_O away from the highest or lowest PEEP step) may be an incentive to consider a wider PEEP range. We also recommend that for intra-patient comparisons, the PEEP range and steps are kept constant if the PEEP trial is repeated over time. Nevertheless, in each clinical setting, physicians should choose the acceptable PEEP range according to the condition of the patients, being aware of the possible issues associated with smaller PEEP windows. We summarize the OD-CL method with step-by-step recommendations in Fig. 2. Other EIT-based methods for individualized PEEP setting different from the decremental PEEP trial have also been explored [42], such as evaluating changes in EELI with PEEP.

### Evaluating the impact of specific procedures/interventions and position

Real-time EIT monitoring can help in the detection of selective bronchial intubation [43, 44], can show derecruitment induced by endotracheal suctioning [45] or broncho-alveolar lavage [46] and may help targeting broncho-alveolar lavage areas [47, 48]. In addition, EIT can be useful to predict the effect of patient positioning. For example, the effect of prone position estimated by EIT could predict the forthcoming gas exchange response [49], while TIV and EELI variations could inform about the effects of both prone and lateral positioning [49–52]. EIT could also guide PEEP settings during prone positioning [53, 54], but the impact on patients’ outcomes remains to be evaluated. Clinicians must be aware that patient mobilization could also lead to changes in TIV and EELI, which should not be interpreted as changes in aeration/ventilation solely.

### Evaluate VILI determinants

The reconstruction of global and regional pressure-impedance curves and of the corresponding regional inflection points may inform about regional overdistension or collapse [55–57]. For instance, regional intra-tidal recruitment, reflecting regional overdistension [58], has been quantified by the regional ventilation delay (RVD) index [59] or concavity indices [60]. EIT-based regional lung opening and closing pressures [61] may be particularly useful in patients with asymmetric lung injury [62]. Currently, bedside availability of these parameters and evidence for their routine use is limited, but may help to dynamically titrate tidal volume based on its regional effects [63]. Clinicians can combine PEEP titration results with tidal volume guidance from EIT for improving lung protection [64], also in addition to other techniques, such as extracorporeal life support (ECCO_2_R or ECMO).

## Clinical applications: spontaneous breathing

EIT may offer valuable insights to safely manage spontaneous breathing in patients with acute hypoxemic respiratory failure who are at risk of P-SILI [65] and guide both weaning/extubation and post-extubation phases. In addition, EIT revealed expiratory muscle recruitment in ARDS patients, evidenced by an increase in EELI after paralysis [66]. EIT has been used for monitoring the effect of different non-invasive respiratory support modalities (i.e., interfaces, modes, settings) and interventions (e.g., changes in body position) on the ventilation distribution and regional mechanics [4, 67, 68]. However, measurements of both respiratory mechanics and EIT are challenging in awake patients [2], due to the large variability in breathing patterns and various movement artifacts. Therefore, only a subset of EIT parameters can be used in this setting (Table 2). To enhance reliability, we suggest careful selection of stable breathing phases and (manual) removal of artifacts if present.

### Monitoring tidal volume and respiratory rate

EIT offers a valuable non-invasive estimate of tidal volume as impedance changes strongly correlate with lung volume changes [16, 69–71]. However, computation of absolute tidal volumes with EIT is more complex, requiring a factor to convert tidal volume to TIV (i.e., *k* = VT/TIV). This factor can be obtained reliably during invasive ventilation, but needs a point calibration with known tidal volumes in non-intubated patients (e.g., via spirometry [24], or simpler calibration bag method [71]), which is often not practical/feasible and guarantees only short-term stability (i.e., only within one recording). Nevertheless, regional TIV distribution assessment is feasible without point calibration and provides potentially useful insights on regional strain.

EIT also allows accurate monitoring of respiratory rate during spontaneous breathing [72]. This facilitates EIT-based estimations of *changes* in minute volume, when combined with TIV [22].

### PEEP titration

Compared to controlled ventilation, PEEP titration in assisted ventilation presents additional challenges due to the variability of respiratory effort, inaccurate measurements of respiratory compliance [73] and hemodynamic fluctuations [74]. Recently, the regional peak inspiratory flow at different PEEP steps was suggested as parameter to quantify regional mechanics during spontaneous breathing [75], and EIT and dynamic transpulmonary pressure were integrated to identify the PEEP that jointly minimizes lung collapse and overdistension during pressure support ventilation [76]. However, limitations and technical challenges remain, related to EIT signal stability, time-consuming offline analysis, need for esophageal manometry and uncertain accuracy in patients with increased airway resistance.

### Weaning

When applied during the spontaneous breathing trial, monitoring regional TIV, de-recruitment and regional inhomogeneities may inform about weaning success [77, 78]. For instance, a reduction in EELI [79–81], higher global inhomogeneity index [22] and pendelluft [82, 83] have been associated with spontaneous breathing trial and/or extubation failure. The predictive role of these parameters to guide weaning remains to be assessed.

### Patient-ventilator interaction

Utilizing the temporal and spatial ventilation distribution information, EIT enables patient-ventilator interaction monitoring and dyssynchronies diagnosis [84]. When combined with flow and/or airway pressure signals, the regional impact of dyssynchronies can be quantified [85, 86], e.g., in terms of ventilation distribution and regional overdistension.

### Pendelluft

Pendelluft, is ‘the volume of gas passing back and forth between two pathways’ [87] and the consequence of inhomogeneities in regional resistances and/or compliances. EIT revealed the occurrence of pendelluft [88] which was correlated with regional inflammation; both could be modulated by PEEP [88]. Negative associations with outcome have been reported [83, 89] but the causal clinical impact remains to be investigated.

EIT is the only technique capable of identifying pendelluft in real-time. Pendelluft measurements during spontaneous breathing have been used to: (1) describe how different ventilatory settings may affect regional time constants, regional resistances and compliances [90], (2) describe the air movement between dependent and non-dependent lung regions during inspiration [85, 91–93], and (3) compute the difference between global and regional redistribution of gas during the respiratory cycles [4, 68, 94]. Various EIT-based definitions/computations have been proposed in different scenarios (Additional file 2).

## Ventilation-perfusion matching

EIT is a promising monitor for regional ventilation-to-perfusion ($\dot{\text{V}}/{\text{Q}}$) distribution: the ratio of regional alveolar ventilation (ml air/min) to regional blood flow (ml blood/min). EIT-based $\dot{\text{V}}/{\text{Q}}$ can be performed easily at the bedside and repeated over time, providing important advantages as compared to other imaging techniques (i.e., CT, SPECT) or to Multiple Inert Gas Elimination Technique (MIGET), which provide only aggregate results of $\dot{\text{V}}/{\text{Q}}$ mismatch. In patients with respiratory failure, EIT $\dot{\text{V}}/{\text{Q}}$ assessment can improve our understanding of gas exchange abnormalities, and allows to evaluate the effects of procedures or ventilator settings on *regional* $\dot{\text{V}}/{\text{Q}}$ (e.g., PEEP adjustment [95], prone positioning [96] and adjunctive therapies, like nitric oxide [97, 98]). Whereas ventilation-related EIT signals are large and reasonably well understood, the $\dot{\text{V}}/{\text{Q}}$ technique is still relatively new in the clinical context and only a preliminary consensus on its use is possible at this time.

**Fig. 3:**
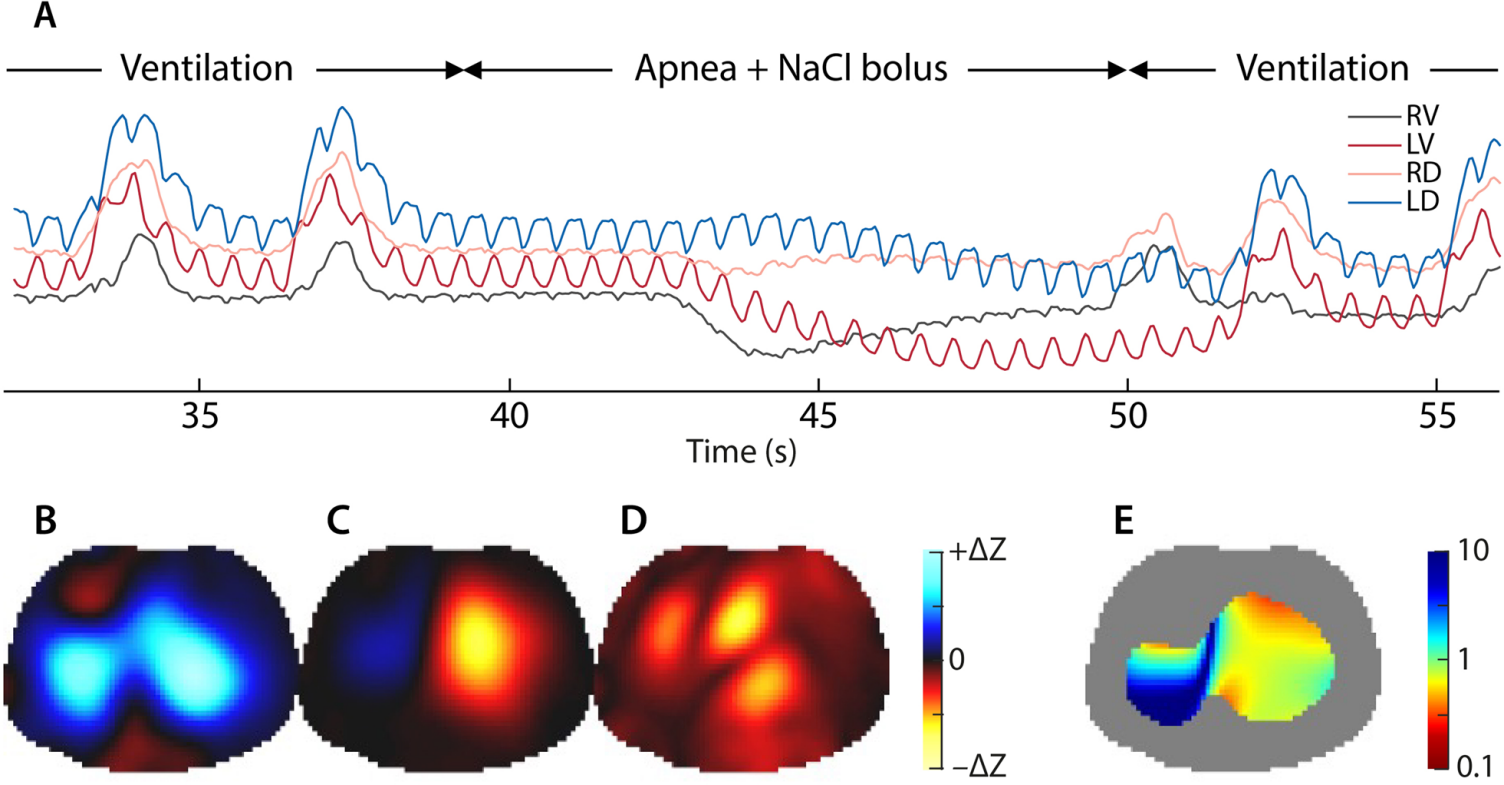
Illustration of ventilation-perfusion assessment by EIT. Waveforms and images from ref. [124] in a patient after pulmonary endarterectomy. **A** EIT waveforms before and during a NaCl bolus and apnea (38–50 s) in four lung quadrants. **B** Tidal ventilation image prior to the NaCl bolus and apnea. **C** Bolus-based perfusion image. The red/yellow indicate the conductivity change during the lung perfusion phase (at approximately 47 s) of the bolus. This is only in the left lung due to the patient’s perfusion defect. **D** Heart rate-filtered EIT image. **E** Relative EIT-$\dot{\text{V}}/{\text{Q}}$ image in the lung region of interest. The color scales used are shown using a log-scale such that 1 indicates mean equal perfusion and ventilation distribution. No consensus for EIT perfusion or $\dot{\text{V}}/{\text{Q}}$ color scales is available. Abbreviations: RV = right ventral, LV = left ventral, RD = right dorsal, LD = left dorsal

Perfusion has been measured via (1) conductivity-contrasting bolus injection (*Bolus technique,* Fig. 3), and (2) through filtering of the pulsatile heart-beat component of EIT signals (*Pulsatility technique*). The bolus technique showed better agreement with both SPECT [99] and PET [100] and is currently the reference for EIT-based $\dot{\text{V}}/{\text{Q}}$ assessment.

### Bolus technique: procedure

Using a central line catheter, a contrast agent, usually 10 mL of hypertonic saline (usually 5–10% NaCl) [100] is injected rapidly (in 1–2 s) during a breath-hold of 8–15 s [101]. EIT images of Q are then calculated by subtraction of the heart pixels [99], whereas the V̇ component is calculated by filtering the pre-apnea ventilation signal to remove heart rate-related components [26] and pixel-by-pixel $\dot{\text{V}}/{\text{Q}}$ matching can be determined [102]. Software to automatically process these EIT data to calculate $\dot{\text{V}}/{\text{Q}}$ images at the bedside is required. If independent measures of minute ventilation and cardiac output are available, a *calibrated* $\dot{\text{V}}/{\text{Q}}$ image can be calculated [103]; else, the EIT-$\dot{\text{V}}/{\text{Q}}$ image is unitless (the *relative* $\dot{\text{V}}/{\text{Q}}$ image).

### Considerations and open questions

A concern may arise regarding the electrolytes load of the saline bolus. Nevertheless, the salt load of the injected bolus is relatively small (10 mL 5% saline bolus corresponds to 0.5 g (8.5 mEq) of NaCl), and no adverse effects of electrolyte disturbances after rapid or repeated bolus administration have been reported to date. However, it is unclear how the 10 mL requirement scales with patient mass, blood volume and cardiac output. Several promising new contrast agents were proposed [104] and are currently under investigation [105]. Recent data demonstrated the feasibility of calculating EIT-derived $\dot{\text{V}}/{\text{Q}}$ without apnea, providing new perspectives on its application when breath-holds are difficult, e.g., during spontaneous breathing [106].

## Standardized EIT reporting in clinical and experimental studies

Considering the impact of the different variables during EIT acquisition, processing and interpretation, we believe that a minimum requirement for EIT reporting would allow traceability, reproducibility and comparability of EIT studies. We therefore propose minimum standards for scientific reporting of EIT in clinical and experimental studies (see Additional file 3). This could provide guidance to researchers and facilitate sustainable implementation of EIT for individualization of ventilation management and stimulate development of standardized open-source analyses pipelines (e.g., ALIVE [107]).

## Future directions

In the last two decades, EIT application has considerably increased, both for technological advances and for a better understanding of respiratory physiology in different settings and under different conditions. Here, we highlight novel future directions that have not yet been discussed above.

### Diagnosing non-ventilated areas

EIT can evaluate regional ventilation, but no specific information can be obtained from the *absence* of ventilation, either determined by physiological (e.g., the ribcage, mediastinum) or pathological (e.g., pleural effusion, overdistension, pneumothorax) entities. Hypo-ventilated lung areas can be identified (i.e., *silent spaces* [108]) but lung contouring is not available on all devices and may require individual adjustments [109]. One open question is therefore how to further characterize non-ventilated areas and how to differentiate tissue properties with EIT. *Absolute EIT* (aEIT), often used in the 1980s and 1990s, evaluates the regional absolute impedance values and not, as the dynamic EIT (dEIT), its changes over time [110]. By allowing measuring different tissues conductivities, aEIT could potentially distinguish between structures, therefore improving the understanding of hypo-ventilated areas.

Devices for simultaneous multi-frequency measurements have already been reported [111–115] but most of the current clinical/commercial EIT devices are based on a single frequency and imaging algorithms for multi-frequency EIT are still under-developed [116, 117]. The reliable detection of atelectasis and its differentiation from pleural effusion, pneumothorax and overdistension according to its spectroscopic properties could be a promising future application of multi-frequency EIT.

### 3D-EIT

EIT monitoring of a single horizontal thoracic slice with a spatial resolution of about 2–3 cm is reasonable for many clinical applications. However, best spatial resolution is limited to the regions located closer to the belt. Movement of the lung and cardiac structures within the electrode plane, e.g., during a PEEP trial, can cause artifacts that can be misinterpreted as recruitment or overdistension. A potential solution is to create EIT images in 3D [118, 119], by placing two belts for simultaneous recording and analyzing them with 3D reconstruction algorithms [120, 121]. 3D-EIT could also improve image quality by correcting for out-of-plane changes in impedance and has the potential of enhancing the reliability of EIT-based $\dot{\text{V}}/{\text{Q}}$ assessment, since lung perfusion is anatomically clearer than in 2D [121]. We encourage further research on 3D-EIT and the development of devices for clinical applications.

### Machine learning applications

In the year 2024 it would be an omission not to mention machine learning in a section on future perspectives of any technology, and EIT makes no exception. Yet only few applications have been published, including deep learning models for image reconstruction [122] and feature extraction for predicting spirometry data and circulatory parameters [123]. We see potential for machine learning to be valuable at multiple stages. For instance: image reconstruction (also for 3D-EIT), lung contour detection, signal filtering, artifact detection and eventually correction, and facilitating in complex analyses. Ideally, such applications should use raw voltage data instead of reconstructed impedance signals since these are already highly processed on-device to serve the human eye at the bedside.

## Conclusion

EIT is a powerful technology for the non-invasive monitoring of regional distribution of ventilation and perfusion, offering real-time and continuous data that can greatly enhance our understanding and management of various respiratory and circulatory conditions. Its application may be especially beneficial for critically ill mechanically ventilated patients, providing insights that can guide the optimization of ventilatory support, assess the effectiveness of therapeutic interventions, and potentially improve outcomes. Through continued innovation, rigorous validation, and collaborative efforts to standardize practices, EIT can become an indispensable tool in the clinician's arsenal, ultimately improving patient care and outcomes in critical care.

## Supplementary Information

Additional file1.

Additional file2.

Additional file3.

## Data Availability

No datasets were generated or analysed during the current study.
