# Supplementary material for: Electrical impedance tomography monitoring in adult ICU patients: state-of-the-art, recommendations for standardized acquisition, processing, and clinical use, and future directions
Source: Crit Care. 2024 Nov 19;28:377. doi: 10.1186/s13054-024-05173-x (PMC11577873; doi:10.1186/s13054-024-05173-x)
Supplement: Supplementary file 1 — Additional file1. [file 13054_2024_5173_MOESM1_ESM.docx]

**Additional file 1** – Different methods for functional lung contouring

| **METHOD** | **DESCRIPTION** | **NOTES** |
| --- | --- | --- |
| **Tidal impedance variation (TIV)** | EIT image pixels with a TIV above a predefined cut-off value are considered ventilated and included in the functional ROI(1) | Cut-off value is typically a percentage of the maximum pixel TIV.  Most commonly used and recommended method for functional ROI selection. |
| **Standard deviation of pixel impedance** | EIT image pixels with a standard deviation of TIV above a predefined cut-off value are considered ventilated and included in the functional ROI.(2) | Cut-off value is typically a percentage of the maximum pixel standard deviation of TIV; may include pixels with negative impedance changes in ROI. |
| **Linear regression coefficient of pixel impedance** | EIT image pixels with a regression coefficient above a predefined cut-off value are considered ventilated and included in the functional ROI. Regression coefficients are derived from linear regression equations between the individual pixel and average global impedance signals.(2) | Cut-off value is typically a percentage of the maximum pixel regression coefficient. |

*Abbreviations: EIT, electrical impedance tomography; ROI, region of interest; TIV, tidal impedance variation.*

**References**

1. Becher T, Vogt B, Kott M, et al. Functional Regions of Interest in Electrical Impedance Tomography: A Secondary Analysis of Two Clinical Studies. PLoS One. 2016;11(3):e0152267.
2. Pulletz S, van Genderingen HR, Schmitz G, et al. Comparison of different methods to define regions of interest for evaluation of regional lung ventilation by EIT. Physiol Meas. 2006;27(5):S115-27.
