# Supplementary material for: Electrical impedance tomography monitoring in adult ICU patients: state-of-the-art, recommendations for standardized acquisition, processing, and clinical use, and future directions
Source: Crit Care. 2024 Nov 19;28:377. doi: 10.1186/s13054-024-05173-x (PMC11577873; doi:10.1186/s13054-024-05173-x)
Supplement: Supplementary file 2 — Additional file2. [file 13054_2024_5173_MOESM2_ESM.docx]

**Additional file 2** – Description of recent definitions of pendelluft in different scenarios/applications.

| **STUDY** | **PATIENTS** | **VENTILATION MODE** | **PENDELLUFT CALCULATION** |
| --- | --- | --- | --- |
| Santini et al.(1) | ARDS | VCV | Pixel-by-pixel difference from zero flow to end inspiration during an inspiratory hold |
| Coppadoro et al.(2) | Weaning  phase | Spontaneaous breathing trial | Sum of inspired volume during expiration + volume expired during expiration |
| Sang et al.(3) | Cases (flail chest, ARDS, COPD) | Non-intubated and ventilated patients | Pixel-by-pixel difference between the global and regional ventilation |
| Cornejo et al.(4) | ARDS | BiPAP | Percentage of the normalized tidal volume that moves from non-dependent to dependent regions during inspiration in each ventilatory cycle |
| Lin et al.(5) | ARDS | Transition from VCV to assisted breathing | Sum of inspired volume during expiration + volume expired during expiration |
| Menga et al.(6) | AHRF | Helmet NIV, helmet CPAP, HFNO | Pixel-by-pixel difference between the global and regional ventilation |
| Grieco et al.(7) | AHRF | HFNO | Pixel-by-pixel difference between the global and regional ventilation |
| Bello et al.(8) | ARDS | PSV, BiPAP | Pixel-by-pixel difference between the global and regional ventilation |
| Arellano et al.(9) | ARDS | NAVA, PAV+, PSV | Percentage of the normalized tidal volume that moves from non-dependent to dependent regions during inspiration in each ventilatory cycle |
| Adler et al.(10) | ARDS  (single case) | PSV | The fraction of reverse flow in each pixel waveform (as an image) or globally (as a single parameter) |
| *Abbreviations: ARDS, Acute Respiratory Distress Syndrome; AHRF, Acute Hypoxemic Respiratory Failure; BiPAP, Biphasic Positive Airway Pressure; CPAP, Continuous Positive Airway Pressure; COPD, Chronic Obstructive Pulmonary Disease; HFNO, High Flow Nasal Oxygen; NAVA, Neurally Adjusted Ventilatory Assist; NIV, Non-Invasive Ventilation; PSV, Pressure Support Ventilation; PAV+, Proportional Assist Ventilation; VCV, Volume-Controlled Ventilation.* | | | |

**References**

1. Santini A, Mauri T, Dalla Corte F, et al. Effects of inspiratory flow on lung stress, pendelluft, and ventilation heterogeneity in ARDS: a physiological study. Crit Care. 2019;23(1):369.

2. Coppadoro A, Grassi A, Giovannoni C, et al. Occurrence of pendelluft under pressure support ventilation in patients who failed a spontaneous breathing trial: an observational study. Ann Intensive Care. 2020;10(1):39.

3. Sang L, Zhao Z, Yun PJ, et al. Qualitative and quantitative assessment of pendelluft: a simple method based on electrical impedance tomography. Ann Transl Med. 2020;8(19):1216.

4. Cornejo RA, Arellano DH, Ruiz-Rudolph P, et al. Inflammatory biomarkers and pendelluft magnitude in ards patients transitioning from controlled to partial support ventilation. Sci Rep. 2022;12(1):20233.

5. Lin WC, Su PF, Chen CW. Pendelluft in patients with acute respiratory distress syndrome during trigger and reverse triggering breaths. Sci Rep. 2023;13(1):22143.

6. Menga LS, Delle Cese L, Rosa T, et al. Respective Effects of Helmet Pressure Support, Continuous Positive Airway Pressure, and Nasal High-Flow in Hypoxemic Respiratory Failure: A Randomized Crossover Clinical Trial. Am J Respir Crit Care Med. 2023;207(10):1310-23.

7. Grieco DL, Delle Cese L, Menga LS, et al. Physiological effects of awake prone position in acute hypoxemic respiratory failure. Crit Care. 2023;27(1):315.

8. Bello G, Giammatteo V, Bisanti A, et al. High vs Low PEEP in Patients With ARDS Exhibiting Intense Inspiratory Effort During Assisted Ventilation: A Randomized Crossover Trial. Chest. 2024;165(6):1392-405.

9. Arellano DH, Brito R, Morais CCA, et al. Pendelluft in hypoxemic patients resuming spontaneous breathing: proportional modes versus pressure support ventilation. Ann Intensive Care. 2023;13(1):131.

10. Adler A, Becher T, Händel C, Frerichs I. Fraction of reverse impedance change (FRIC): a quantitative electrical impedance tomography measure of intrapulmonary pendelluft. Physiol Meas. 2024 Sep 25.
