# Supplementary material for: Electrical impedance tomography monitoring in adult ICU patients: state-of-the-art, recommendations for standardized acquisition, processing, and clinical use, and future directions
Source: Crit Care. 2024 Nov 19;28:377. doi: 10.1186/s13054-024-05173-x (PMC11577873; doi:10.1186/s13054-024-05173-x)
Supplement: Supplementary file 3 — Additional file3. [file 13054_2024_5173_MOESM3_ESM.docx]

**Additional file 3** – Checklist for standardized EIT reporting

We recommend standardized reporting of methods used for acquisition, processing and analyses of EIT data, since all hardware and software methods as well as applied maneuvers impact subsequent results. Therefore, to enhance reproducibility and generalizability of EIT studies in human subjects, we stimulate transparent reporting of all methods. The following checklist could serve as a guidance.

|  | RECOMMENDED CONTENT | PRESENT IN REPORT (YES/NO, PAGE) |
| --- | --- | --- |
| 1 | EIT device used (brand, model, software version) |  |
| 2 | Number of electrodes, contact agent (if any) |  |
| 3 | Frame rate |  |
| 4 | Belt positioning and size selection |  |
| 5 | Patient position |  |
| 6 | Procedure for coping with pleural drains in the examination area, if appropriate. |  |
| 7 | Reconstruction algorithm (on-device/other). If other, sufficient details on image reconstruction method. |  |
| 8 | Measures for prevention and handling of measurement artifacts |  |
| 9 | Filtering (on-device/custom), including which method |  |
| 10 | Lung contouring and ROI selection method |  |
| 11 | Maneuvers performed, if applicable |  |
| 12 | Method for selection of stable periods |  |
| 13 | Parameter extraction (breath-by-breath, averaged breaths?) |  |
| 14 | Handling of longitudinal measures, if appropriate |  |
| 15 | OD-CL procedure (ventilatory mode, settings, calculation), if appropriate |  |
| 16 | Method for perfusion imaging (technique, bolus, processing), if appropriate |  |
